# Supplementary figures and images for: deepAMPNet: a novel antimicrobial peptide predictor employing AlphaFold2 predicted structures and a bi-directional long short-term memory protein language model
Source: PeerJ. 2024 Jul 19;12:e17729. doi: 10.7717/peerj.17729 (PMC11262304; doi:10.7717/peerj.17729)

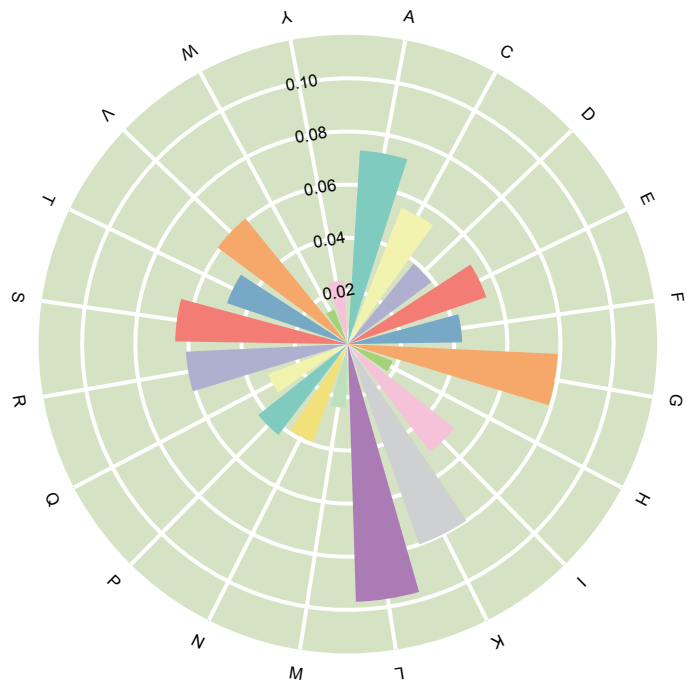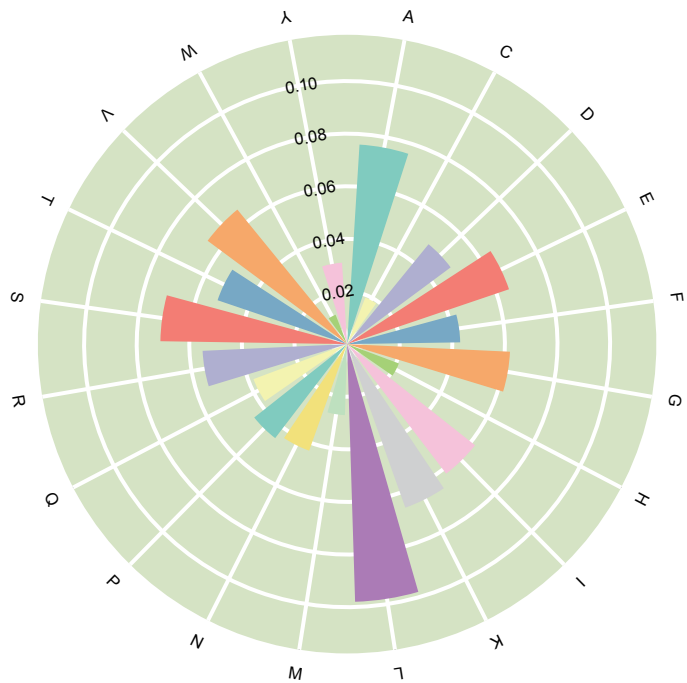

Supplement: Supplemental Information 1 [file peerj-12-17729-s001.pdf]

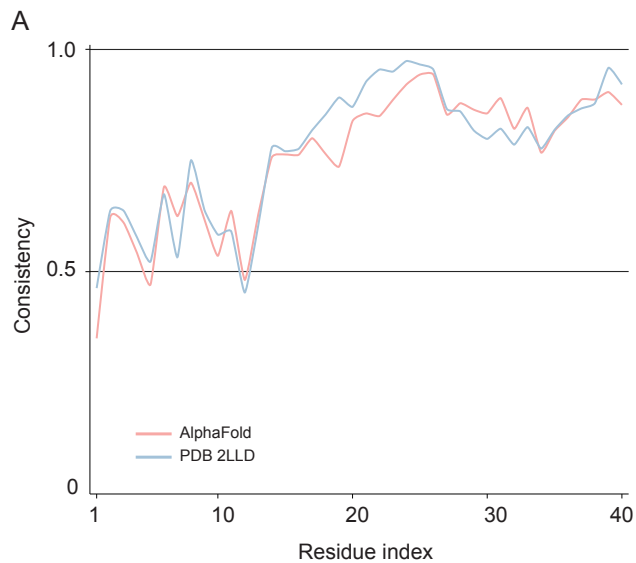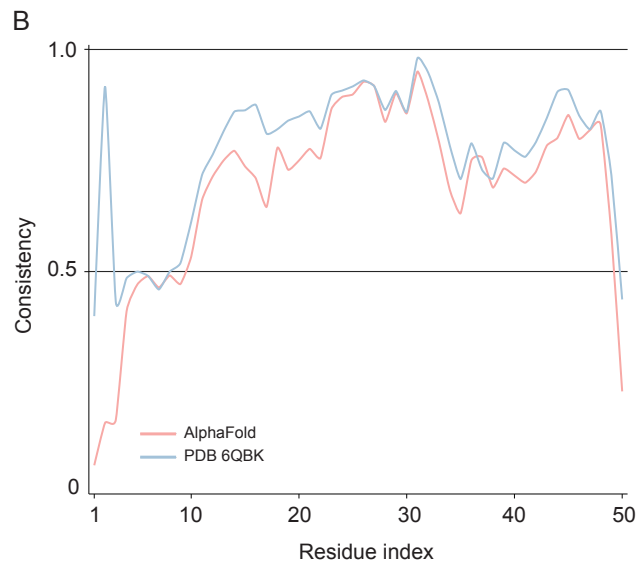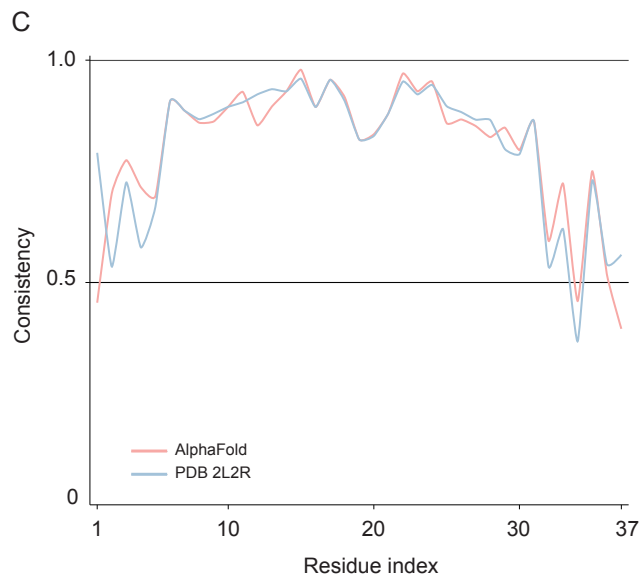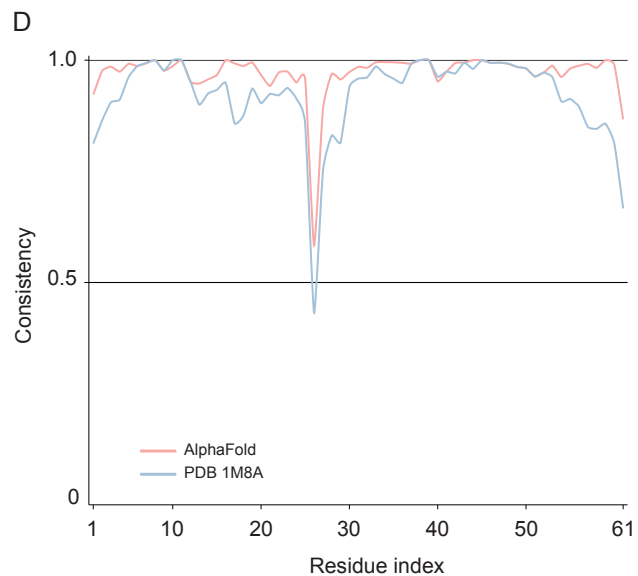

Supplement: Supplemental Information 2 [file peerj-12-17729-s002.pdf]

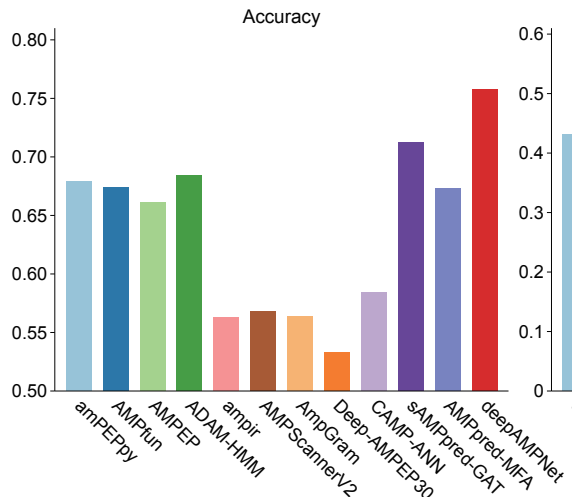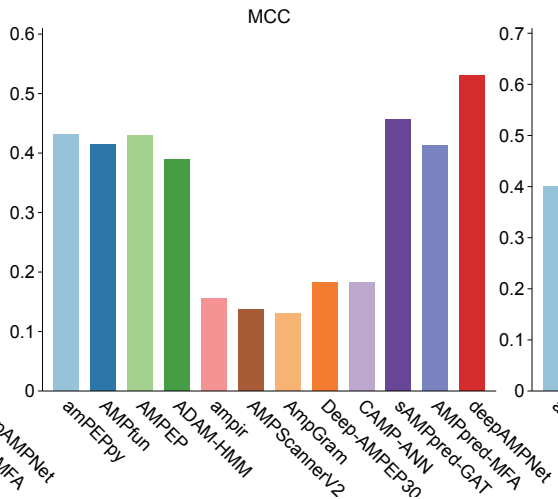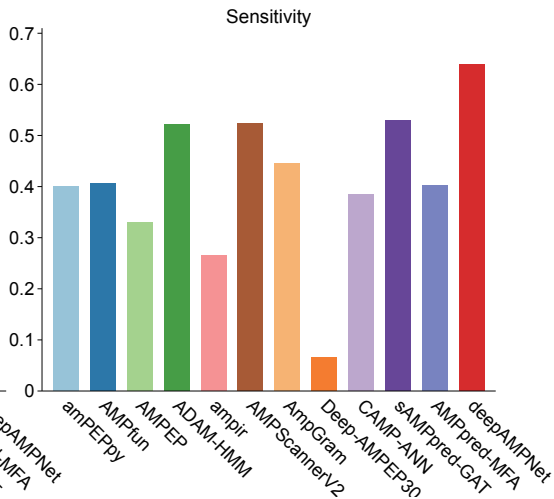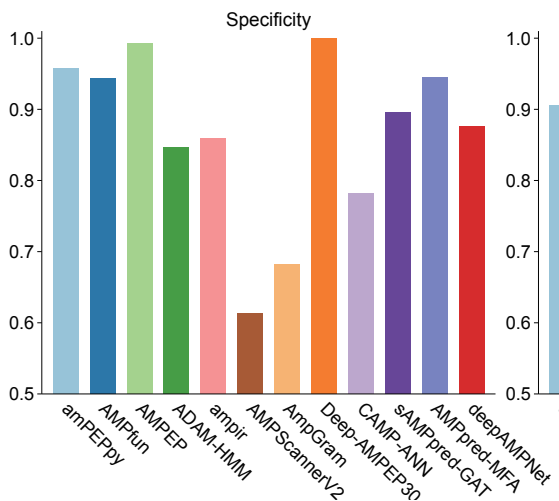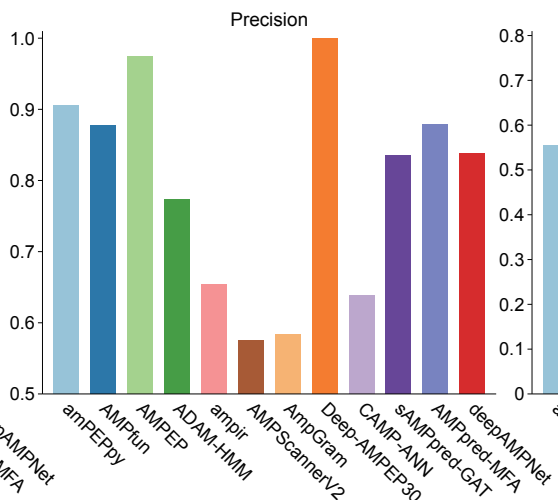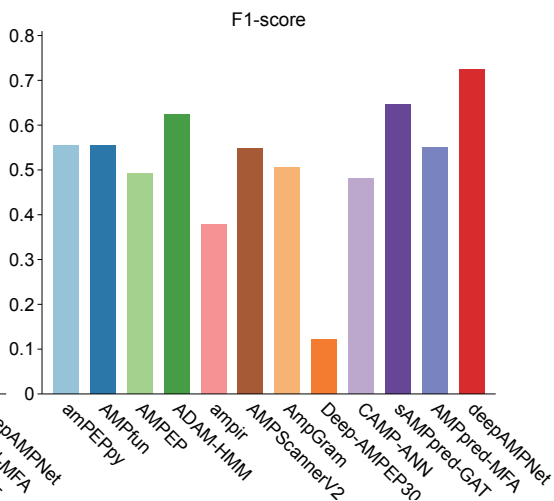

Supplement: Supplemental Information 3 [file peerj-12-17729-s003.pdf]
